# The Impact of Global Warming and Anoxia on Marine Benthic Community Dynamics: an Example from the Toarcian (Early Jurassic)

**DOI:** 10.1371/journal.pone.0056255

**Published:** 2013-02-14

**Authors:** Silvia Danise, Richard J. Twitchett, Crispin T. S. Little, Marie-Emilie Clémence

**Affiliations:** 1 School of Geography, Earth and Environmental Sciences, Plymouth University, Plymouth, United Kingdom; 2 School of Earth and Environment, University of Leeds, Leeds, United Kingdom; Ludwig-Maximilians-Universität München, Germany

## Abstract

The Pliensbachian-Toarcian (Early Jurassic) fossil record is an archive of natural data of benthic community response to global warming and marine long-term hypoxia and anoxia. In the early Toarcian mean temperatures increased by the same order of magnitude as that predicted for the near future; laminated, organic-rich, black shales were deposited in many shallow water epicontinental basins; and a biotic crisis occurred in the marine realm, with the extinction of approximately 5% of families and 26% of genera. High-resolution quantitative abundance data of benthic invertebrates were collected from the Cleveland Basin (North Yorkshire, UK), and analysed with multivariate statistical methods to detect how the fauna responded to environmental changes during the early Toarcian. Twelve biofacies were identified. Their changes through time closely resemble the pattern of faunal degradation and recovery observed in modern habitats affected by anoxia. All four successional stages of community structure recorded in modern studies are recognised in the fossil data (i.e. Stage III: climax; II: transitional; I: pioneer; 0: highly disturbed). Two main faunal turnover events occurred: (i) at the onset of anoxia, with the extinction of most benthic species and the survival of a few adapted to thrive in low-oxygen conditions (Stages I to 0) and (ii) in the recovery, when newly evolved species colonized the re-oxygenated soft sediments and the path of recovery did not retrace of pattern of ecological degradation (Stages I to II). The ordination of samples coupled with sedimentological and palaeotemperature proxy data indicate that the onset of anoxia and the extinction horizon coincide with both a rise in temperature and sea level. Our study of how faunal associations co-vary with long and short term sea level and temperature changes has implications for predicting the long-term effects of “dead zones” in modern oceans.

## Introduction

The expansion of oxygen minimum “dead zones” in modern oceans is at the top of the list of emerging environmental challenges [1]. General circulation models predict that climate change will directly deplete oceanic dissolved oxygen levels by increasing stratification and warming, as well as indirectly by causing changes in rainfall patterns, nutrient run-off and shelf eutrophication; all of which will increase the areas affected by hypoxia (dissolved oxygen ≤2 ml/l, [2]) and anoxia [3]–[5]. One of the major goals of marine biologists is thus to understand the effect of hypoxia on marine organisms at different temporal and spatial scales [6]. Hypoxia can affect coastal areas seasonally, periodically or inter-annually [3], and depending upon its duration and severity, it may take years or even decades for recovery of the original community composition [7]. Although the impact of low-oxygen conditions on ecosystems is relatively well understood through field and experimental studies, the processes of recovery are still poorly understood and the experimental assessment of hypoxia remains challenging [7]. Furthermore, the pattern of species reappearance post-event may follow a different trajectory to that of species loss, resulting in a hysteresis-like response of biodiversity to the alleviation of hypoxia [3].

Hypoxia can occur at a variety of temporal and spatial scales. Only the smallest temporal and spatial scales may be readily observed or recreated experimentally [2], [8]–[9], and it is unclear whether these results are applicable at larger scales. Data from the largest temporal, spatial and ecological scales can, however, be sourced from the fossil record, which provides an archive of natural data from a number of past episodes of climatic and environmental change [10]–[12]. A detailed fossil record with good temporal resolution that spans past climate change events can help in forecasting future ecosystem changes, especially if predicted climate changes move outside the parameters experienced by modern ecosystems and into regimes known only from the deeper geological record [13].

Rocks spanning the Pliensbachian-Toarcian interval of the Early Jurassic (∼185-181 Ma) are an archive of natural data from one of these past episodes of global warming and anoxia. Temperatures are estimated to have increased by 2–3.5°C in subtropical areas [14] and 6–8°C [15]–[16] at higher latitudes, values which are similar to the increases forecast for the end of the 21^st^ century [17]. In many early Toarcian shallow, epicontinental basins worldwide, laminated, organic-rich, black shales, which formed under anoxic conditions, were deposited [18]–[19]. Widespread anoxia and seawater palaeotemperature rise are also associated with a 400–800% increase in global weathering rates [20]. In some localities, anoxia temporarily spread into the lower photic zone, together with euxinia, as indicated by the presence of biomarkers of green sulphur bacteria in some black shales [21]–[23].

Marine ecosystems were adversely affected by these climate-driven environmental changes, and early Toarcian strata record a major extinction of marine organisms, particularly amongst the infaunal benthos [24]–[32]. Evidence of an extinction event has been reported in western Europe (e.g., [24], [33]–[35]), South America [36]–[37], Siberia [38] and northern Africa [39]. Although definitive cause-effect relationships are not yet established, the extinction has either been linked to the development of widespread anoxia [27], [37], [40]–[41], or to global warming [32], [42].

Despite the importance of this event in the early evolution of modern marine ecosystems, and as a potential analogue for current environmental concerns, there is no quantitative palaeoecological study of community-level changes in marine macro-invertebrates through the Pliensbachian-Toarcian interval. Previous studies have mostly involved the collection of presence/absence data of fossil macro-invertebrates, in order to identify extinction horizons by analysing the stratigraphic distribution of species [24]–[25], [31], [43]–[44] and to compare with global extinction-recovery models [45]. Other quantitative palaeoecological studies have analysed changes in the body sizes of selected fossil molluscs [46]. In this study, we apply multivariate statistical analyses of fossil abundance data to explore how benthic macro-invertebrates responded to Pliensbachian-Toarcian environmental changes. Although these techniques have been proven to be excellent tools in understanding how marine assemblages change through time in relation to past environmental changes (see [47]), they have been rarely applied to extinction events (e.g., [48]–[49]).

The main aim of this study is to analyse how benthic communities changed through an interval of past global warming characterized by the development of widespread hypoxic and anoxic conditions in marine settings. In particular we aim to assess (i) how alpha and beta diversity (in terms of faunal turnover) changed through the studied interval; (ii) how benthic communities responded to sea level, oxygen concentrations and temperature variations; and (iii) how this fossil event compares to the recorded patterns of faunal succession in modern communities affected by hypoxic and anoxic conditions.

## Materials and Methods

### Geological setting

This study is focused on the Pliensbachian-Toarcian sections of the northern Cleveland Basin (UK) because of its well-preserved fossil record of macro-invertebrates, and the abundance of published geochemical, sedimentological and stratigraphical data, which are useful in interpreting environmental change. In the Early Jurassic, the Cleveland Basin was situated at a palaeolatitude of 30–40°N [14] and formed part of a system of shallow epicontinental seas and small extensional tectonic basins connected to the North Sea (Figure S1) [50]. The upper Pliensbachian Cleveland Ironstone Formation is dominated by coarse grained siliciclastic mudstones, with some calcareous concretionary units, very fine-grained muddy sandstones and sideritic and chamositic, shelly ironstones that cap overall upward-coarsening successions [51]–[53]. It is subdivided into the lower Penny Nab Member and the upper Kettleness Member that are separated by a basinwide unconformity [33], [51]. The presence of gutter casts, wave ripple laminae and distal tempestites indicate that the Cleveland Ironstone Formation was deposited on a storm-dominated marine shelf [53]–[54].

The overlying lower Toarcian Whitby Mudstone Formation is subdivided into three members. The lower Grey Shale Member consists of light to dark grey silty mudstones with horizons of calcareous and sideritic concretions [55]. The majority of the Grey Shale Member is extensively bioturbated [56]–[57], and although it was traditionally interpreted as being deposited in a low-energy, outer shelf setting (e.g., [52]), the recent identification of small ripples, wave-enhanced mudflows and micro-scale hummocky cross-stratification indicates that occasionally high energy conditions developed at the sediment-water interface [43], [57]. Furthermore, hydrogen index values and pyrite-framboid sizes indicate that oxygen-restricted conditions began to develop in the upper part of the Grey Shale Member, which led to the development of an oxygen minimum zone in the water column [27], [43], [58]. The lower part of the Mulgrave Shale Member (the “Jet Rock”; *exaratum* subzone) consists of dark grey, organic-rich shales, laminated on a millimetre-scale, which contain regular horizons of centimetre to metre scale calcareous concretions. Total organic carbon content is very high, ranging between 5% and 15% [59]–[60], and the abundance of pyrite framboids indicates that anoxic and sulphidic conditions characterised the water column and sediments for most, but not all, of the time [43]. In the upper part of the Mulgrave Shale Member (*falciferum* subzone) laminations are less pronounced and the content of organic matter is lower [60]. The Alum Shale Member (*bifrons* and *variabilis* zones) consists of dark grey, non-bituminous shales, with abundant calcareous nodules [59], and represents a renewed influx of fine-grained terrigenous siliciclastic sediments into the basin, under more oxygenated conditions [61].

### Ethics statement

No permits were required for the described study, which complied with local guidelines in relation to responsible collecting from foreshore exposures.

### Sampling

Coastal sections were sampled at Staithes, Port Mulgrave, Runswick Bay, Kettleness and Saltwick Bay (Figure S1). Rock exposure in these sections is close to 100% and correlation between them is possible via numerous marker beds of carbonate nodules, sideritic concretions, and distinctive lithologies [55], [59], [62]. The total dataset consists of 154 samples of quantitative abundance data of benthic macro-invertebrates, and the average stratigraphic resolution of samples is less than 0.5 metres (Figure S2). Of these, 91 samples were collected by CTSL [63], and the remaining 63 were collected by SD, RJT and MEC during 2012 from foreshore exposures. CTSL's samples [63] were collected every metre, on average, through the study interval, apart from at the top of the Grey Shale Member where the samples were collected at a decimetre scale. At each sample point, all the benthic macro-invertebrate fossils found in the field were counted and species abundance was recorded. Representative specimens were taken back to the laboratory for identification. The 2012 samples were collected every 1.5 metres on average, through the study interval, and a standard quantity of 3 kg of bulk rock was collected at each sample point. Samples were mechanically disaggregated in the laboratory with the use of sharp chisels, and all recognisable macrofossils were counted and identified. The total, combined dataset comprises 12283 individuals of 56 species, and includes abundance data for bivalves, gastropods, scaphopods, brachiopods, echinoderms (crinoids) and serpulids, which were identified at the finest taxonomic level possible (Table S1). For bivalves and brachiopods, the number of individuals was obtained by adding together the number of articulated specimens and the number of disarticulated valves. The number of gastropod individuals was equated to the number of individual apices, and each discrete cluster of crinoid ossicles was counted as one individual. Reference bed numbers, ammonite zones and subzones follow Howarth [55], [59], [62]; lithostratigraphy follows Howard [51] and Rawson & Wright [52].

### Analytical Methods

For each sample alpha diversity was measured using the Simpson index of diversity (1-d), taxonomic diversity (Δ) and taxonomic distinctness (Δ*), which are appropriate measures of diversity for species abundance data when sample size is not homogeneous [64]. The Simpson index of diversity ranges from zero (one taxon dominates the community completely) to one (all taxa are equally present), and it represents the probability that two individuals randomly selected from a sample will belong to different species. Δ and Δ* measure the relatedness of the species within a sample. They are based not just on species abundances, but also on taxonomic relationships of the taxa making up the dataset [65]. Δ is the average “taxonomic distance apart” of each pair of individuals in one sample. If the taxonomic tree comprises a single-level hierarchy, Δ resembles the Simpson index of diversity. Δ*, on the other hand, measures the expected taxonomic distance apart of any two individuals chosen at random from one sample, provided those two individuals are not from the same species. To calculate Δ and Δ*, information on the taxonomic hierarchy (genus, family, superfamily, order, class, phylum) of each species was added to the dataset to create a taxonomic tree showing the relatedness of the species in each sample (Table S2). Following [65], constant step lengths were used between successive taxonomic levels and the weight given to each branch length was standardized so that the longest path in each tree is 100. For all the three indices, the curve was smoothed using the 3-point moving average function [66].

For multivariate elaboration, species occurring in only one sample were removed, and samples containing less than 20 individuals were excluded. The resulting culled data set includes 47 species, 115 samples and 11936 individuals (97.2% of the original specimens). Percentage data were used because the volume of each sample was not consistent and the absolute numbers of individuals are thus not comparable between samples [64]. Some samples were strongly dominated by few species, and so the percentage data were square-root transformed to de-emphasise the influence of the most abundant taxa.

Nonmetric multidimensional scaling (nMDS) with the Bray Curtis similarity matrix, and detrended correspondence analysis (DCA) are among the best ordination methods for detecting ecological gradients in modern and ancient marine environments (e.g., [47], [64], [67]). Although the results of the two ordinations methods were similar, DCA compressed variation on axis 2, such that the clouds of data points resembled a triangle (Figure S3). In agreement with other authors ([e.g., [68]), we therefore prefer nMDS as the best method to explore species distributions in this study. The stress criterion [69] was used to evaluate goodness-of-fit for the final nMDS model. Because the scaling and orientation of axes in nMDS are arbitrary, the configuration was rotated to have the greatest variation along the first axis, and sample scores were used as univariate scores for the nMDS axes.

Analysis of similarity (ANOSIM) was carried out to test the degree of differences between *a priori* groups of samples [64]. The resulting pair-wise R-values give an absolute measure of separation between the groups, on a scale of zero (indistinguishable) to one (no similarity between groups). When the analysis was carried out to test the difference between samples collected with the two different sampling methods, the null hypothesis (no difference between the two *a priori* groups of samples) was retained (R = 0.003; p = 0.324). This indicates that samples do not group according to the two different sampling methods and therefore the two datasets are comparable. The homogenous nature of the combined dataset can also be confirmed by nMDS ordination (Figure S4).

Hierarchical agglomerative clustering (CLUSTER analysis), with the unweighted pair-group average cluster model, was applied to recognise those species that tend to co-occur in samples and to group together samples of similar taxonomic composition. The similarity profile test (SIMPROF) was applied to determine significant difference between the clusters [64]. This technique is a permutation test of the null hypothesis that a specified sets of samples, which are not *a priori* divided into groups, do not differ from each other in multivariate structure. A detailed description of the methods can be found in [70]. In our specific case, 1000 permutations were applied to calculate a mean similarity profile, 999 simulated profiles were generated, and the chosen significance level is 5%. The resulting clusters of samples were analysed through a similarity percentages analysis (SIMPER) to determine which species were responsible for the greatest similarity within groups; see [71] for details of the SIMPER methodology. The species identified by the analysis as typifying each group are (i) those that occur at a consistent abundance in each sample within the group, so that the standard deviation of their contribution (Sd) is low, and (ii) those where the ratio between the average similarity within the group (Sim) and Sd is low [71]. This method enabled the identification of groups of samples that contain a similar suite of taxa in similar proportions (i.e. “biofacies” *sensu* [72]), and also to identify their characteristic species. For those species, life habit information were added, which include tiering (surficial, semi-, shallow-, and deep-infaunal) and feeding mechanism (suspension, surface deposit, mining, chemosymbiotic). Diversity, cluster and ordination analyses were performed with the software PRIMER 6 [73].

Although lithology it is not an absolute palaeoenvironmental indicator, it may be used as a general guide to palaeoenvironment as parameters such as grain size broadly correlate with depositional energy and, frequently, with water depth [74]. The lithologies recorded in this study are oolitic ironstone, very fine sandstone, siltstone, silty-mudstone, mudstone, clay-rich weakly-laminated mudstone, clay-rich laminated mudstone. The presence of laminations in mudstones reflects the absence of bioturbating animals and hence is used as an indirect indicator of reduced oxygen conditions [75].

**Figure 4:**
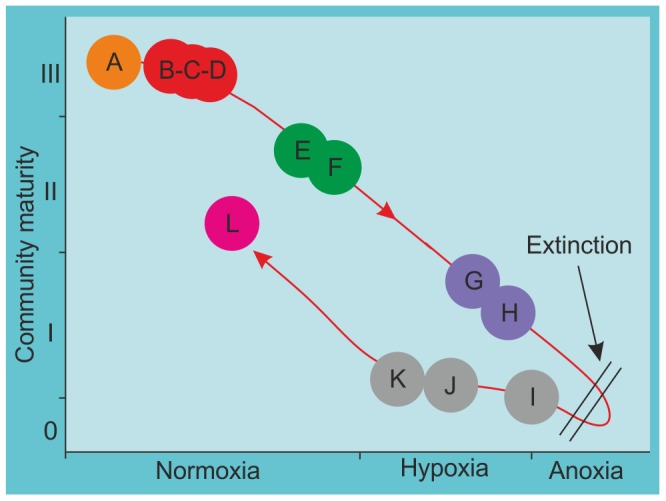
Model of the benthic community response to hypoxia and anoxia during the late Pliensbachian and early Toarcian. Community maturity stages: III, undisturbed/climax communities; II, transitional communities; I, pioneer/disturbed communities; 0, grossly disturbed communities. Letters refer to biofacies in Figures 2 and 3. Note: the recovery path from Stage 0 to II is different from the decline from Stage III to 0 because of species extinction after prolonged hypoxia and anoxia. The post-anoxia Stage II community (biofacies L) has a different taxonomic composition from the former ones (E, F: Stage II) due to the origination and immigration of new species in the intervening time period. Figure modified from [3].

Ordination values were plotted against the stratigraphic log and compared with relative changes in sea levels and palaeotemperature. A curve of relative sea level changes for the Cleveland Basin was derived by Hesselbo and Jenkyns ([76]: figure 11) and Hesselbo ([77]: figure 4). A δ^18^O curve based on belemnite, bivalve and brachiopod calcite was derived from literature data published on the Cleveland Basin [78]–[80], and fitted with the LOESS smoothing function ([66]; smooth 0.02). Trends in δ^18^O were interpreted in terms of relative, rather than absolute, changes in palaeotemperature, because other factors such as species-specific effects (e.g., [81]), especially in fossil coleoids [82], and an unknown initial seawater chemistry [83] can affect the interpretation of absolute temperatures.

## Results

### Alpha diversity

**Figure 1:**
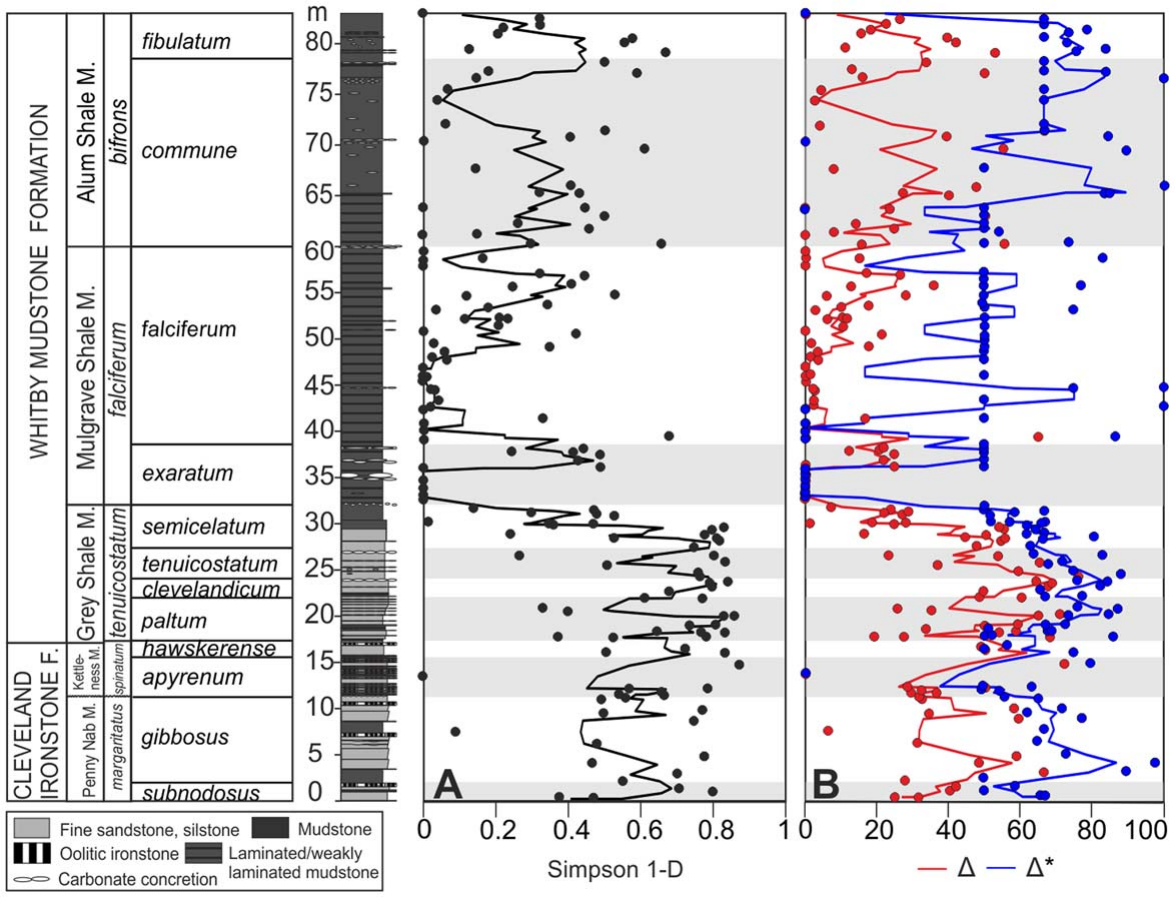
Late Pliensbachian to early Toarcian variations in the alpha diversity of benthic ecosystems of the Cleveland Basin. (A) Simpson index of diversity (1-d). (B) Taxonomic diversity (Δ) in red and taxonomic distinctness (Δ*) in blue. Each point represents the measured value for each sample; the lines were smoothed using the 3-point moving average function. F = Formation; M = Member. Italics indicate ammonite zones and subzones (see text for details). The white and grey bars delimit ammonite subzones. For more details on the lithology see Figure S2.

The Simpson index of diversity (1-d) ranges from 0.4 to 0.8 in the *margaritatus*, *spinatum* and *tenuicostatum* zones, indicating the benthic assemblages have an even distribution (Figure 1). In the upper part of the Grey Shales (the *semicelatum* subzone), the index falls to zero over a very short stratigraphic distance, which indicates a change to communities dominated by a single taxon. In the *falciferum* and *bifrons* zones, the value of the index fluctuates between 0 and 0.4, but, although average values are higher in the *bifrons* zone, never returns to the same evenness distribution as in the lower part of the section. Taxonomic diversity (Δ) records a similar trend to the Simpson index (Figure 1), with the highest values in the lower part of the section, and zero values (i.e. assemblages comprising a single species) being recorded in the *exaratum* subzone. The *tenuicostatum* zone decline in Δ, however, begins in the *clevelandicum* subzone, earlier than the Simpson index. Trends in Δ* are similar to Δ until the *exaratum* subzone (Figure 1). Thereafter, values are moderately high in the *falciferum* subzone, and return to values comparable to those of the lower part of the section in the *commune* subzone.

### Changes in faunal taxonomic composition

**Figure 2:**
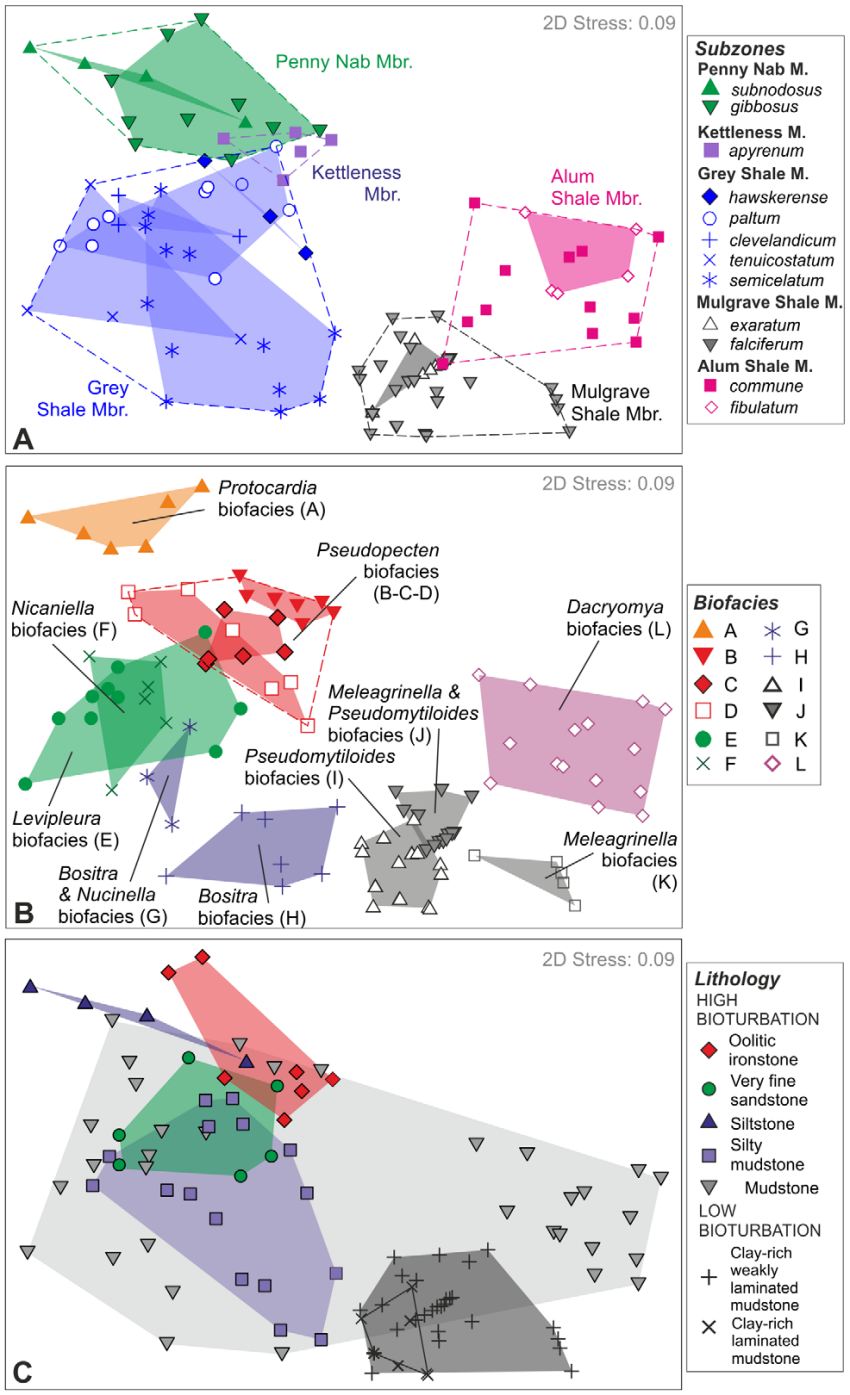
nMDS ordination of samples. (A) Samples labelled and grouped according to their stratigraphic unit and ammonite subzone (see Figure 1). (B) Samples grouped according to the biofacies identified by the CLUSTER analysis (see text for details). (C) Samples grouped according to lithology. Note, because the scaling and orientation of axes in nMDS are arbitrary, the configuration was rotated to have the greatest variation along the first axis.

The results of nMDS ordination demonstrate that samples group according to their stratigraphic units (i.e. members), with little or no overlap between most of them (Figure 2A). The stress value for the nMDS ordination is 0.09, which gives confidence that the two dimensional plot is an accurate representation of the sample relationships. ANOSIM confirms that there is a significant difference in faunal composition between samples belonging to different members (R = 0.77, p<0.01) (Table 1). Members are defined on the basis of their lithological characteristics, each representing a discrete palaeoenvironmental setting. When stratigraphically (i.e. temporally) consecutive members are compared (Table 1), the ANOSIM pairwise test shows that samples from the Penny Nab and Kettleness Members are similar (R = 0.16, p = 0.09), as are samples from the Kettleness and Grey Shale Members (R = 0.20, p = 0.04), even though these latter two are lithologically distinct and are placed in different formations. In contrast, samples from the Grey Shale and Mulgrave Shale members are very different (R = 0.77, p<0.01), as are samples from the Mulgrave Shale and Alum Shale Members (R = 0.78, p<0.01).

**Table 1:** ANOSIM pairwise test between samples from consecutive stratigraphic units.

Sample statistic (Global R): 0.769					
Significance level of sample statistic: 0.0001					
			R	p	Statistical decision
Penny Nab Mbr.	v	Kettleness Mbr.	0.16	0.09	s
Kettleness Mbr.	v	Grey Shale Mbr.	0.20	0.04	s
Grey Shale Mbr.	v	Mulgrave Shale Mbr.	0.77	0.0001	s
Mulgrave Shale Mbr.	v	Alum Shale Mbr.	0.78	0.0001	s

A finer temporal resolution is provided by subzones, which are here defined by faunal changes in the nekton (i.e. ammonites) without reference to lithology. There is a good degree of overlap in the ordination space between groups of samples (subzones) belonging to the same stratigraphic unit (Member) (Figure 2A). This is also shown by the ANOSIM test (Table 2): R values among groups of samples belonging to the same stratigraphic unit are always equal to or less than 0.50 (when statistically significant), which means that groups strongly overlap. When stratigraphically consecutive subzones are compared, R values are very low (<0.3) until the *tenuicostatum* subzone. The greatest differences are between samples from the *semicelatum* and the *exaratum* subzones, and from the *falciferum* and *commune* subzones (R = 0.68, p<0.01 in both cases; in bold in Table 2).

**Table 2:** ANOSIM pairwaise test between subzones.

Sample statistic (Global R): 0.71. Significance level of sample statistic: 0.0001						
A. ANOSIM pairwise test between subzones within the same stratigraphic unit						
				R	p	Statistical decision
Penny Nab Mbr.	*subnodosus*	v	*gibbosus*	0.26	0.05	s
Kettleness Mbr.	*apyrenum*	v	*hawskerense*	0.28	0.13	s
Grey Shale Mbr.	*paltum*	v	*clevelandicum*	−0.09	0.66	ns
	*paltum*	v	*tenuicostatum*	0.50	0.00	s
	*paltum*	v	*semicelatum*	0.46	0.00	s
	*clevelandicum*	v	*tenuicostatum*	0.11	0.37	ns
	*clevelandicum*	v	*semicelatum*	0.29	0.01	s
	*tenuicostatum*	v	*semicelatum*	0.19	0.06	s
Mulgrave Shale Mbr.	*exaratum*	v	*falciferum*	−0.12	0.98	ns
Alum Shale Mbr.	*commune*	v	*fibulatum*	−0.03	0.55	ns

B. ANOSIM pairwise Test between stratigraphically consecutive subzones						
				R	p	Statistical decision
	*subnodosus*	v	*gibbosus*	0.26	0.05	s
	*gibbosus*	v	*apyrenum*	0.12	0.16	s
	*apyrenum*	v	*hawskerense*	0.28	0.13	s
	*hawskerense*	v	*paltum*	0.29	0.06	s
	*paltum*	v	*clevelandicum*	−0.09	0.66	ns
	*clevelandicum*	v	*tenuicostatum*	0.11	0.37	ns
	*tenuicostatum*	v	*semicelatum*	0.19	0.06	s
	***semicelatum***	**v**	***exaratum***	**0.68**	**0.0001**	**s**
	*exaratum*	v	*falciferum*	−0.12	0.98	ns
	***falciferum***	**v**	***commune***	**0.68**	**0.0001**	**s**
	*commune*	v	*fibulatum*	−0.03	0.55	ns

### Biofacies changes and environmental gradients

**Figure 3:**
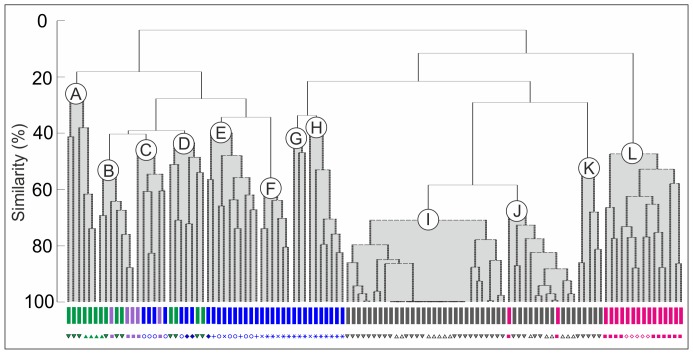
CLUSTER analysis. The CLUSTER analysis, together with the SIMPROF test, identified 12 groups of samples (coloured in grey) which are statistically distinct. The thick black lines indicate at which similarity levels the clusters are grouped together by the SIMPROF test. The 12 groups have been interpreted as different benthic biofacies. Coloured bars and symbols indicate the different stratigraphic units and ammonite subzones each sample belongs to (colours and symbols as in Figure 2A).

Bivalves dominate the dataset, representing 96.4% of the total number of individuals. They are followed by brachiopods (1.2%) and gastropods (1.2%), and all the other categories together (scaphopods, echinoids and serpulids) are less than 1% in abundance. Cluster analysis shows that at low values of similarity (<20%) samples are subdivided into two main clusters (Figure 3). One cluster comprises samples from the Cleveland Ironstone Formation and most samples from the Grey Shale Member, and the other includes all the samples of the Mulgrave Shale and Alum Shale Members, along with samples from the top of the Grey Shale Member (GS20 and GS30–GS41). The SIMPROF permutation test resulted in the discrimination of twelve distinct groups (or biofacies; labelled A to L in Figure 3). This method, together with the SIMPER analysis of similarity (Table 3), enabled identification of the characteristic species within each group, and definition of the twelve different biofacies.

**Table 3:** SIMPER analysis on biofacies and species life habit information.

Species	Sim/SD	Contrib.%	Cum.%	Tiering	Feeding
**Biofacies A**	Average similarity: 37.72				
*Protocadia truncata*	1.2	40.98	40.98	SH-I	SF
**Biofacies B**	Average similarity: 61.94				
*Pseudopecten equivalvis*	5.03	34.8	34.8	SU	SF
*Palmoxytoma cygnipes*	5.22	27.11	61.91	SU	SF
**Biofacies C**	Average similarity: 55.09				
*Pseudopecten equivalvis*	4.26	18.02	18.02	SU	SF
*Plicatula spinosa*	4.29	12.71	30.73	SU	SF
*Tetrarhynchia tetraedra*	1.32	8.45	39.18	SU	SF
*Pseudolimea acuticostata*	1.3	8.01	47.18	SU	SF
*Oxytoma inequivalve*	1.24	7.44	54.62	SU	SF
*Lobothyris punctata*	1.33	7.29	61.92	SU	SF
*Pleuromya costata*	1.35	7.03	68.94	DP-I	SF
*Protocadia truncata*	1.32	6.95	75.89	SH-I	SF
*Modiolus scalprum*	1.3	6.49	82.39	SM-I	SF
**Biofacies D**	Average similarity: 48.34				
*Pseudopecten equivalvis*	3.68	44.41	44.41	SU	SF
*Pseudolimea acuticostata*	6.32	35.13	79.54	SU	SF
**Biofacies E**	Average similarity: 49.56				
*Levipleura blainvillei*	1.6	19.58	19.58	SU	DT
*Nicaniella striatosulcata*	1.71	18.06	37.64	SH-I	SF
*Paleonucula navis*	1.06	16.59	54.23	SH-I	DP
*Tetrarhynchia tetraedra*	1.24	13.06	67.3	SU	SF
*Pseudolimea acuticostata*	1.24	11.57	78.87	SU	SF
**Biofacies F**	Average similarity: 64.25				
*Nicaniella striatosulcata*	7.88	22.05	22.05	SH-I	SF
*Pseudolimea acuticostata*	4.08	19.1	41.16	SU	SF
*Parainoceramus* sp.	1.29	10.48	51.64	SU	SF
*Grammatodon insons*	1.25	10.13	61.77	SU	SF
*Pseudopecten equivalvis*	1.28	9.5	71.26	SU	SF
*Nuculana sp.*	1.31	8.93	80.19	SH-I	DP
**Biofacies G**	Average similarity: 44.20				
*Bositra radiata*	2.16	49.32	49.32	SU	SF
*Pseudolimea acuticostata*	33.07	31.28	80.59	SU	SF
*Nucinella* sp.	0.58	10.35	90.94	SH-I	CH
**Biofacies H**	Average similarity: 58.75				
*Bositra radiata*	2.67	63.91	63.91	SU	SF
*Pseudomytiloides dubius*	1.41	32.57	96.48	SU	SF
**Biofacies I**	Average similarity: 82.45				
*Pseudomytiloides dubius*	5.39	96.71	96.71	SU	SF
**Biofacies J**	Average similarity: 80.61				
*Meleagrinella substriata*	4.63	51.7	51.7	SU	SF
*Pseudomytiloides dubius*	4.54	47.78	99.47	SU	SF
**Biofacies K**	Average similarity: 62.23				
*Meleagrinella substriata*	2.65	81.94	81.94	SU	SF
**Biofacies L**	Average similarity: 56.79				
*Dacryomya ovum*	2.87	67.57	67.57	SH-I	DP

Only species with a Sim/SD ratio >1 are included. Abbreviations: average contribution/standard deviation (Sim/Sd), % species contribution (Contrib. %), cumulation of % species contribution (Cum.%). Tiering: surficial (SU), semi-infaunal (SM-I), shallow-infaunal (SH-I), deep-infaunal (DP-I). Feeding: suspension (SF), detritus (DT), deposit (DP), chemosymbiotic (CH). Underlined taxa were used as reference names of the biofacies in the text. *Nucinella* sp. is the only shown species with Sim/SD ratio <1.

In the lower part of the section biofacies are not restricted to single Members (Figure 2A–B, 3). The Penny Nab, Kettleness and Grey Shale Members share biofacies A to D. Biofacies E to H are only found in the Grey Shale Member but are not restricted to single biozones. In contrast, in the higher part of the section biofacies are mainly restricted to single stratigraphic units. Biofacies I to K are typical of the Mulgrave Shale Member, with two exceptions (Figure 3), and biofacies L is only found in the Alum Shale Member (Figure 3).

The main axis of the ordination (nMDS1) mostly reflects temporal changes through the studied section, with samples from the Penny Nab, Kettleness and Grey Shale Members on the left side of the diagram, and samples from the Mulgrave and Alum Shales on the right (Figure 2A). Ordination results are expected to reflect temporal gradients in species composition if, as in the present study, there is a significant temporal turnover in the fauna [68]. The distribution of samples along axis 2 (nMDS2), however, can be correlated instead to palaeoenvironmental gradients, in particular to changes in lithology and oxygen levels. Broadly, grain size decreases from the top (oolitic ironstones, very fine sandstones and siltstones) to the bottom (clay-rich mudstones) of the ordination, and if bioturbation is used as a proxy of the degree of dissolved oxygen around the sediment-water interface, then oxygenation also decreases in the same direction, with the most oxygen deficient (laminated and weakly laminated) sediments at the bottom of the diagram (Figure 2C).

## Discussion

### Extinction and faunal turnover

The diversity analyses show that during deposition of the Cleveland Ironstone Formation and of the majority of the Grey Shale Member a diverse benthic population was present (Figure 1). Diversity abruptly declined from the base of the *semicelatum* subzone, reaching minimum values at the base of the *exaratum* subzone. This main drop in diversity corresponds to the extinction horizon recognised by Little [25], and to the highest extinction level of Harries and Little [45] and Caswell et al. [31]. It coincides with evidence for gradual stratification of the marine system which led to the development of an oxygen-minimum zone in the Cleveland Basin and with the onset of long-term hypoxia to anoxia [43], [58], [83].

Both taxonomic richness and distinctness, however, record a progressive decline from the earlier *clevelandicum* subzone, which indicates that the benthic community underwent progressively increasing stress before the *semicelatum* subzone. In fact, a decrease in the hierarchical level of diversity of species is expected with increasing levels of environmental perturbation [84]. The environmental stress could have been caused by the gradual development of reducing conditions within the sediments together with reduction in the amount of dissolved oxygen in the bottom water from the top of the *clevelandicum* subzone [56], probably linked to a marked increase in terrestrial sedimentary input because of sea level rise [27]. The earliest Toarcian also registered two short periods of water mass restriction and significant pyrite formation, represented by thin (∼10 cm) laminated black shale facies (“sulphur bands”) [58].

Minimum values of alpha diversity persisted for most of the *exaratum* subzone, and coincide with the interval of maximum water mass restriction and stratification in the basin, deposition of organic-rich sediments and high runoff rates [27], [58], [85]. Alpha diversity starts to increase again from the higher part of the *exaratum* subzones, where water mass restriction was less intense [58]. The oscillations of alpha diversity in this interval probably reflect periodic oxygenation events which allowed the epifauna to briefly colonize the substrate. From the *falciferum* zone, taxonomic distinctness is relatively high compared with taxonomic diversity, indicating that the assemblages comprise relatively few, taxonomically unrelated species. Bivalves dominate the samples in term of abundance, with either rare brachiopods (e.g., *Discinisca papyracea*, *Lingula longovicnensis*), echinoderms (e.g., *Pentacrinites* sp.) or gastropods (e.g., *Procerithium* sp.) also occurring periodically. A relaxation of stressed conditions from the *bifrons* zone is testified by a general increase in alpha diversity.

One method of estimating beta diversity, in terms of compositional turnover, is to use the results of the ANOSIM test (see [48], [86]). A mass extinction event can be considered as the end-member along a continuum in turnover caused by environmental disturbance. Turnover pulses are in fact strongly correlated with climatic shifts, changes in ocean circulation, and fluctuation in sea level, suggesting a strong link between environmental and biotic change [47]. The ANOSIM test highlights the occurrence of two main turnover events in the studied section (Table 1, 2). The first occurs between the *semicelatum* and the *exaratum* subzones and coincides with the large drop in alpha diversity of the extinction event. The second occurs between the *falciferum* and the *commune* subzones, corresponding to a large increase in taxonomic distinctness (Figure 1) and coinciding with the onset of the recovery interval [45]. High turnover rates may indicate recovery as well as crisis, and in this study show that once oxygen levels rose sufficiently a new suite of benthic species moved in to colonize the substrate.

### Patterns of faunal degradation

The changes of biofacies through time in the studied section represent a sequence of community replacement (*sensu* [87]), in which palaeocommunity changes are driven by temporal changes in the environment. Environmental forcing of biotic change is now widely recognized to occur over a range of geographical and temporal scales throughout the Phanerozoic, although cause-end-effect relationships are not always well understood ([88] and references therein).

The sequence of palaeocommunity replacement through time in the present study may also be compared with the pattern of successional stages that have been recognised in modern ecosystems affected by anoxia, despite obvious differences in temporal scale. In modern ecosystems affected by anoxia, four successional stages in benthic community structure along a gradient of declining oxygen concentrations have been recognised [2]–[3], [89]. Stage III represents undisturbed, climax communities in normally oxygenated conditions. Transitional communities (Stage II) occur in systems that experience periodic hypoxia, and pioneer or disturbed communities (Stage I) occur in systems with persistent hypoxia. With increasing hypoxia the system moves to grossly disturbed communities (Stage 0), until all the macrofauna disappear at the onset stable and persistent anoxia. Equivalents to these successional stages can be recognised in this study (Figure 4).

#### Stage III (biofacies A–D)

The *Protocardia truncata* (A) and *Pseudopecten equivalvis* biofacies (B, C, D) represent undisturbed communities, characterise the lower part of the studied section, and indicate a high energy, clear water and well oxygenated environment. Biofacies A has a large compositional variability, as shown by the low SIMPER average similarity value (37.7: Table 3). The most common species was an active shallow burrower, the cardiid *P. truncata*, probably adapted to thrive in low turbidity, nutrient rich settings [90]. *P. equivalvis* was a eurytopic epifaunal species that thrived best in high energy, oxygenated settings, where its growth was favoured by an increased supply of suspended food [91]. The diversity of lifestyles within these biofacies, including the presence of semi-, shallow- and deep-infaunal suspension feeding bivalves, is diagnostic of normal benthic oxygen levels [92].

#### Stage II (biofacies E–F)

In the *Nicaniella striatosulcata* and *Levipleura blainvillei* biofacies deposit feeders become more frequent (e.g., *Paleonucula navis*, *Nuculana* sp.), as do small infaunal suspension feeding bivalves (e.g., *N. striatosulcata*), with respect to biofacies of Stage III. There is a reduction in the diversity of deep infaunal suspension feeders, which suggests a decrease in sediment oxygen levels. There is also an increase in the abundance of the opportunistic detritus feeding gastropod *Levipleura blainvillei*, which was probably feeding on organic material, microbes and bacteria [93]. These changes in the community structure probably reflect transitional communities that experienced a general decrease in water energy and an increase of nutrient supply in the soft sediments of the Grey Shale Member. This increase in nutrient supply coincides with high surface productivity of marine phytoplankton registered in the surface waters of the lower part of the *tenuicostatum* zone [27], and with periods of periodic anoxia (“sulphur bands”, [58]) in the Cleveland Basin.

#### Stage I (biofacies G–H)

The two biofacies dominated by *Bositra radiata* (G, H) in the *semicelatum* subzone mark a dramatic change in faunal composition and reflect disturbed communities living just before the extinction. Most authors agree that *Bositra radiata* was an epifaunal suspension feeding bivalve adapted to live in low-oxygen conditions ([31] and references therein), even though a nektoplanktonic mode of life was hypothesized by others [94]–[95]. Biofacies G is also characterized by the very short stratigraphic occurrence of the nucinellid species *Nucinella* sp., defined by Wignall et al. [43] as a “disaster” species. Symbionts were recently discovered in one modern *Nucinella* species, and chemosymbiosis inferred for the entire family [96], so that the appearance of *Nucinella* sp. could indicate an increase in sulphide levels in the sediments. In modern marine environments chemosymbiotic infaunal bivalves in symbiosis with sulphur oxidizing bacteria, e.g., the lucinids, thrive in sulphide-rich sediments. However, the presence of some oxygen is a prerequisite, because free-living animals can only tolerate anoxia for limited time periods and the symbionts require oxygen to combine with sulphide to produce energy [97]. Thus, the subsequent disappearance of *Nucinella* sp. probably indicates the shift to more persistent sediment anoxia.

#### Stage 0 (biofacies I)

Biofacies I is a grossly disturbed community represented solely by *Pseudomytiloides dubius*, and occurs mostly in laminated, oxygen deficient, sediments of the *exaratum* and *falciferum* subzones. The palaeoecology of *P. dubius* has been long debated, with some authors suggesting a pseudoplanktonic and others a benthic mode of life (see [31] for a review). As suggested by Röhl et al. [98] for the early Toarcian Posidonia Shale of South West Germany, benthic colonization by *P. dubius* was probably episodically possible for some months to years when the redox boundary fluctuated near the sediment-water interface. At other times, the anoxic lower water column prevented all macrobenthic colonization. In modern oxygen minimum zones, the mytiloid *Amygdalum anoxicolum*, which has planktotrophic larval development and is tolerant to low-oxygen conditions [99]–[100], provides a likely extant analogue for *P. dubius*.

### Patterns of faunal recovery

Intense water mass restriction continued in the Cleveland Basin for about 900 ka in the upper *semicelatum* and *exaratum* subzone, with a frequency of water mass renewal estimated as being between 4 and 40 ka [58]. Successively, in the *falciferum* and *commune* subzones restriction was less severe, with a much higher renewal frequency of between 10 and 130 years [58]. In these intermittent oxygenated intervals communities of Stages 0 to I could colonize the soft bottom, depending on oxygen availability, until oxygenated conditions were re-established and communities moved to Stage II (Figure 4).

#### Stage I (biofacies J–K)

Biofacies J and K mainly occur in the weakly laminated mudstones of the *falciferum* zone. They formed under less restricted water mass conditions than biofacies I, or in longer periods of elevated oxygen content which enabled recurring benthic colonization over several years, as in the early Toarcian Posidonia Shale of South West Germany [98]. They are characterized by *Meleagrinella substriata* and *Pseudomytiloides dubius*, opportunistic species tolerant to low oxygen levels that survived the extinction event, and by *Bositra buchi*, a new species that evolved from surviving lineages [45].

#### Stage II (biofacies L)

Biofacies (L) signals the return to oxygenated, bioturbated sediments in the Alum Shale Member, and is mostly characterized by newly evolved species, such as the shallow infaunal deposit feeding bivalve *Dacryomya ovum* and the infaunal brachiopod *Lingularia longovicensis*. Notwithstanding the fact that species composition of the returning communities is different from the pre-extinction ones, they are structured in a very similar way. In fact, Stage II biofacies before and after the extinction are all characterized by shallow infaunal suspension and deposit feeding molluscs, indicative of oxygenated, high nutrient conditions. The presence of an unconformity above the Alum Shale Member means that complete recovery to Stage III is not recorded in the study area.

As observed by Harries & Little [45], the post-anoxia, re-colonizing benthic community comprises opportunistic species tolerant to low-oxygen levels which were present in the basin before the extinction (e.g., *Pseudomytiloides dubius*, *Meleagrinella substriata*), but mostly new species from surviving lineages (e.g., the bivalves *Bositra buchi*, *Gresslya donaciformis, Dacryomya ovum* and the brachiopods *Discinisca papyracea* and *Lingularia longovicensis*). Post-extinction species largely belong to the same genera as the species becoming extinct, and their appearance is therefore due to the origination of new species and/or immigration of closely related taxa. Aberhan [101] concluded that within bivalves, origination rates exceed migration rates for the early Toarcian, and that either (i) extinction survivors in oxygenated refugia may have evolved into new species which then migrated into vacated areas when conditions improved, or (ii) species from regions such as southern Europe evolved into new species after immigration.

In the studied section, local extinction among deposit- and suspension-feeding infaunal bivalves, semi-infaunal bivalves, gastropods and brachiopods was 100% at the species level, whereas epifaunal bivalves and crinoids, were less affected by the extinction (respectively 78% and 50% species level local extinction) [45]. Most of these species also became regionally extinct [33], whereas others migrated into nearby basins where environmental conditions were more hospitable. For instance, the gastropods *Levipleura blainvillei* and *Ptychomphalus expansus*, which disappear from the Cleveland Basin after the extinction, occur in the *falciferum* zone of the adjacent East Midlands Shelf [102], and the deep infaunal suspension feeding bivalve *Pholadomya ambigua* occurs in the *falciferum* zone of central Spain [103]. Others, like the pectinids *Pseudopecten equivalvis* and *Camptonectes subulatus*, are found in middle-upper Toarcian sediments of Spain and Greenland [91].

### Faunal replacement and environmental changes

The pattern of faunal degradation and recovery discussed above demonstrates that oxygen concentration played an important role in marine community dynamics through the studied interval. However, sea level and palaeotemperature also varied through the section, and likely had a role in controlling faunal change. All three factors co-vary to some extent, as water temperature is a key factor controlling sea-level changes [104] and has a strong modulating effect on oxygen thresholds for hypoxia [5].

Ordination can extract high resolution palaeoenvironmental signals from quantitative data, especially when palaeoecological analyses are integrated within a sequence stratigraphic framework ([47] and references therein). Water depth is in general the main indirect factor controlling the distribution of marine organisms, even though other factors like substrate consistency, grain size and oxygen and nutrient concentrations are known to have a significant effect on assemblages [68], [105]–[106]. In the present study, however, high faunal turnover through the section due to extinction, origination and migration, means that the distribution of samples along the main axis of ordination (nMDS1) is mainly controlled by time. Samples are on average younger from left to right of the ordination (Figure 2A). In contrast, the ordination of samples along axis 2 of the nMDS plot appears to be controlled by both oxygen content and lithology (Figure 2C). Assuming that in general finer grain sizes correspond to deeper water conditions, plotting the nMDS axis 2 sample scores against the stratigraphic section provide a reconstruction of the water depth history.

**Figure 5:**
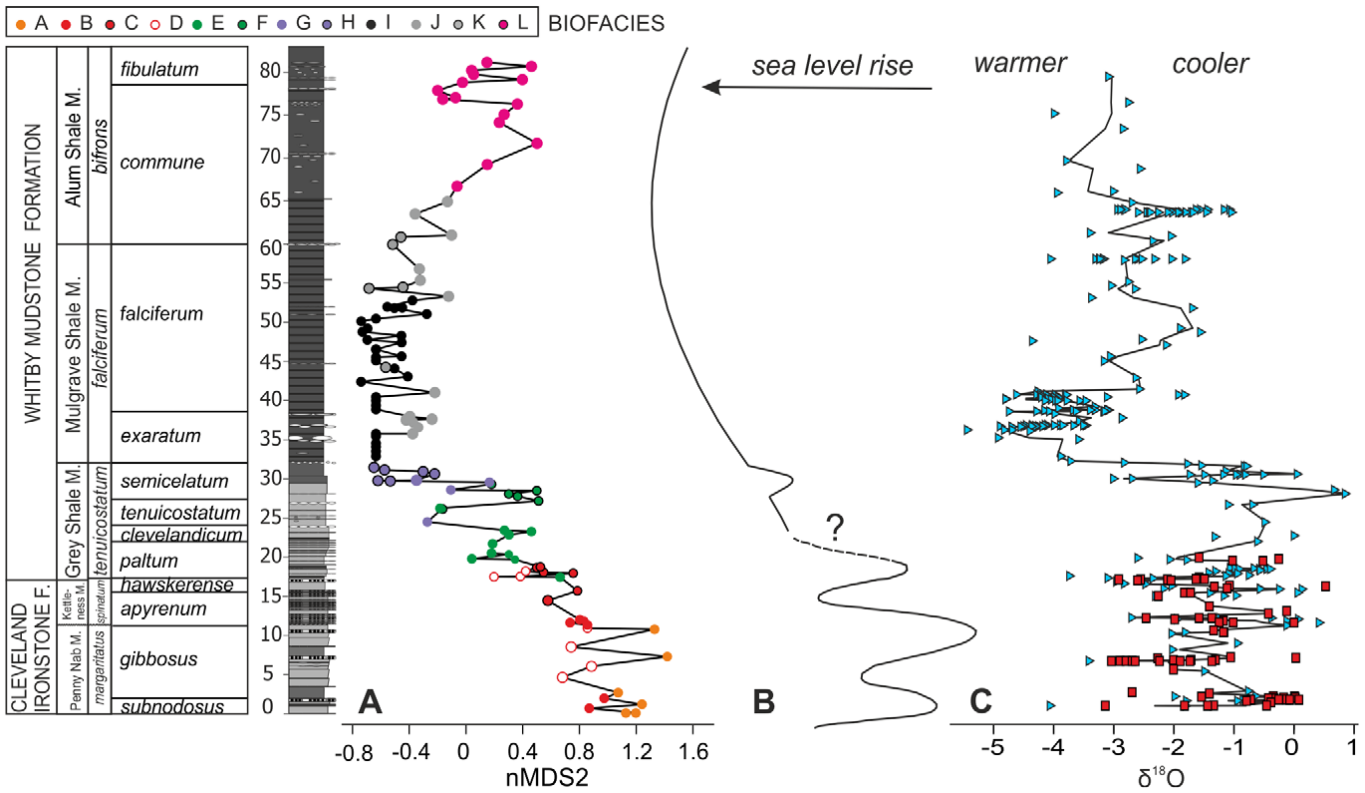
Comparison of relative sealevel, palaeotemperature and nMDS axis 2 sample scores through the studied section. (A) nMDS axis 2 sample scores, each point represents one sample. Colours refer to the different biofacies. (B) Relative sea level curve compiled from [71] and [72]. (C) δ^18^O isotope values from belemnites, brachiopods and bivalve calcite interpreted as reflecting relative palaeotemperatures; data from [73]–[75]. Triangles: belemnites, squares: brachiopods and bivalves. Stratigraphy as in Figure 1.

The resulting curve (Figure 5A) shows strong similarities to the relative sea level curve derived from independent sedimentological and sequence stratigraphic observations in the Cleveland Basin (Figure 5B). Both the large scale late Pliensbachian-early Toarcian eustatic sea level transgression [76]–[77], [107] and smaller scale depositional cycles [53], [76] can be clearly identified. Three distinct short-term transgressive-regressive cycles in the Penny Nab Member, each capped by deposition of an ironstone bed, are followed by a longer term deepening trend from the base of the Kettleness Member to the lower part of the Mulgrave Shale Member (*exaratum* subzone). Superimposed on this longer term deepening are a number of transgressive-regressive cycles at the base of, and within, the Grey Shale Member up to the *semicelatum* subzone. In the Mulgrave Shale Member the new curve shows minor fluctuations without substantial changes in the relative water depth, followed by shallowing within the Alum Shale Member.

In the lower part of the section (Cleveland Ironstone Formation and bottom part of the Grey Shale Member) there is no evidence for oxygen restriction, and changes in biofacies are strongly linked to changes in relative water depth. The distribution along nMDS2 of biofacies A to D, which correspond to Stage III communities that lived under normally oxygenated conditions, seems to be mainly controlled by water depth: biofacies A only occurs in the shallowest sediments, whereas biofacies B, C and D are associated with deeper water (Figure 5A–B). In the Grey Shale Member, the observed pattern of faunal degradation runs in parallel with increasing water depth: Stage II communities (Biofacies E–F), which lived under normally oxygenated conditions, are shallower than the oxygen-restricted Stage I communities (Biofacies G–H) (Figure 5A–B). It is difficult to disentangle the role played by water depth and oxygenation for those samples which occur in oxygen-restricted, fine sediments (clay-rich mudstones), as the decrease in oxygen content is concomitant with the increase in water depth. Biofacies I, J and K (Stages 0 and I) occur in the most oxygen-restricted and, presumably, deepest water conditions. Finally, the return to normally oxygenated conditions, marked by biofacies L (Stage II), is accompanied by a relative decrease in water depth (Figure 5A–B).

The apparent transgressive-regressive cycles in the ordination-based curve (Figure 5A) also correspond closely to warming-cooling cycles recorded by the δ^18^O isotopes derived from belemnites, bivalves and brachiopod calcite from the Cleveland Basin, especially in the Cleveland Ironstone Formation and lower Whitby Mudstone Formation (Figure 5C: data from [78]–[80]). As expected, relative sea level rises mainly coincide with warming trends and sea level falls with cooling. In the Cleveland Ironstone Formation, the coarser-grained shallow water facies (sandstone and ironstones) and their resulting communities were deposited under cooler conditions, and the deeper water facies (mudstones) and fauna relate to warmer conditions (cf. [80]). Even within mudstone-dominated Grey Shale Member with relatively limited grainsize variation, similar cycles in the biofacies curve are evident (Figure 5A,C).

The good match between relative sea level and palaeotemperature changes indicates that temperature was an important factor controlling sea level variations and, as a consequence, community structure through time in the Cleveland Basin. A cause-and-effect relationship between the early Toarcian progressive warming and the concomitant loss of species has been previously hypothesized for late Pliensbachian-early Toarcian sections of Spain [32], [108]. Our data show that the greatest relative increase in water temperature occurs at the top of the Grey Shale Member (*semicelatum* subzone), coinciding with a sharp sea level rise, and culminating in the extinction horizon (Figure 5).

Whereas in the Cleveland Basin, anoxia clearly affected benthic community dynamics, its role in the global extinction event is equivocal. Gómez et al. [108] concluded that the early Toarcian extinction was not primarily caused by anoxia, because it occurred synchronously in western European basins that record anoxic conditions as well as in oxygenated settings of the European and North African platforms that do not. As the development of widespread anoxia was a likely consequence of global warming, it is difficult to separate these two factors in the Cleveland Basin record.

### Implications for predicting marine community response to climate change

The fossil record is an archive that offers important insights into ecosystem dynamics at a range of temporal scales that are of real benefit in helping to constrain predictions of ecological and evolutionary responses to present and future climate change [109]. This potential of the fossil record is strengthened when patterns of recorded change are similar to those of smaller scale studies, despite differences in starting conditions, species composition and temporal scale.

Modern ecosystems affected by severe hypoxia show a non-linear, hysteresis-like progression of successional communities, where the pattern of recovery is different to the pattern of degradation [3]. This happens because the recovery is usually of longer duration than ecosystem degradation, may be interrupted by recurrent hypoxic events, and involves opportunistic re-colonization by pioneers before the return to oxygenated conditions and the restoration of the normal community [7], [89], [110]. Depending on all these factors, communities impacted by hypoxia can take years to recover to the original community composition and density as those present before the hypoxic event [7].

Similarly, in our case-study, ecological recovery took longer than the pattern of degradation; was hindered by recurrent hypoxic events; and started with the re-colonization by opportunistic taxa. Stressed conditions increased from the *clevelandicum* subzone, and culminated with the development anoxia at the top of the *semicelatum* subzone. While the extinction interval was relatively short, being restricted to the upper part of the *semicelatum* subzone (<100 ka, following [79]), maximum water mass restriction lasted longer, for about 900 ka, to the end of the *exaratum* subzone [58]. Afterwards, in the *falciferum* and *commune* subzones (duration of 500 ka according to [79]), short oxygenated events alternated with restricted conditions. About 200 ka after the end of anoxia (middle *commune*-*fibulatum* subzone) the recovery of the benthic fauna was still not complete. Thus, even though the events described in this study occurred over almost 5 Ma [79], sea level and palaeotemperatures varied together with oxygen levels, and faunal changes involved evolutionary, not just ecological processes, the general patterns of benthic community change were comparable to those predicted for modern communities affected by much smaller scale anoxic episodes. This raises the possibility that processes described here would also be recorded at intervening scales, like those predicted for the next century.

## Conclusions

This study demonstrate how high quality, quantitative, palaeoecological analyses of benthic invertebrates integrated within a sequence stratigraphic and environmental framework can advance our understanding of the response of past ecosystems to large-scale environmental changes.

- Analyses of alpha and beta diversity demonstrate that a net decrease in diversity coupled with high faunal turnover characterizes the extinction horizon at the top of the Grey Shale Member (*semicelatum* subzone). A second faunal turnover event occurred between the Mulgrave and Alum Shale members, when newly evolved taxa replaced the impoverished fauna during recovery.
- Twelve biofacies were identified, representing a community replacement sequence that is similar to the successional stages recognised in modern ecosystems affected by anoxia. Benthic communities moved from an undisturbed, climax community (Biofacies A to D, Stage III), through gradually increasing stressed conditions, characterized by episodic hypoxia, increased nutrient supply, and increased oxygen stress in the sediments (Biofacies E–F, Stage II). At the onset of long-term hypoxia only disturbed, pioneer communities were present (Biofacies G–H, Stage I), with the occurrence of the possibly chemosynthetic bivalve *Nucinella* sp. marking a short interval where sulphidic conditions persisted in the sediments, overlain by poorly oxygenated bottom waters. During maximum water mass restriction, colonization by grossly disturbed communities comprising a single species was possible for brief intervals of slightly elevated oxygen levels (Biofacies I, Stage 0). Under slightly less restricted conditions, the community was a little more diverse (Biofacies J–K, Stage I). Under renewed oxygenated conditions new species colonized the sediments (Biofacies L, Stage II).
- Ordination of samples coupled with sedimentological and palaeotemperature proxy data demonstrated that variations in benthic communities correlate with long and short term sea level and temperature changes. The onset of anoxia and the extinction horizon coincide with both a rise in palaeotemperature and sea level. Unlike the situation in modern settings affected by anoxia, this study demonstrates that when the duration of warming-related hypoxia and intense anoxia is long enough (∼900 ka in this case), and extinction occurs, the benthic system will move to a new stable state characterised by newly evolved species.

## Supporting Information

Figure S1 **Location map of the study area.** (A) Schematic structural map of the Cleveland Basin, modified from [50]. MWH: Market Weighton High. (B) Location of the studied sections on the North Yorkshire coast.
(PDF)

Figure S2 **Detailed stratigraphic section.** Stratigraphic log of the studied composite section with details of ammonite zones and subzones, lithology, bed numbers (following [55], [59], [62]), location of the main shell beds and of the collected samples and sampled localities (in blue).
(PDF)

Figure S3 **Comparison of nMDS and DCA ordination of samples.** (A) nMDS ordination with labels as samples numbers. (B) nMDS ordination with labels as bed numbers. (C) DCA ordination with labels as sample numbers. Note that nMDS and DCA produce similar clusters of samples, however the DCA ordination has a triangular shape derived by the compression of values on axis 2.
(PDF)

Figure S4 **nMDS plots showing the distribution of samples collected with two different sampling methods.** (A) nMDS ordination of axis 1 versus axis 2. (B) nMDS ordination of axis 2 versus axis 3. In green samples collected by SD, RJT and MEC; in blue samples collected by CTSL. Note, even though collected with two different sampling methods, the samples show a very high degree of overlap. This, together with the results of the ANOSIM test (R = 0.003, p = 0.324) discussed in the text, indicates that the dataset is homogenous.
(PDF)

Table S1 **Species-level abundance distribution of the collected samples.** Taxonomic composition and abundance of each collected sample. Additional information include the associated lithostratigraphic unit, the subzone, the bed number, the stratigraphic height and the sampling method.
(XLSX)

Table S2 **Taxonomic information for the species listed in Table S1.** Information on the taxonomic hierarchy (genus, family, superfamily, order, class, phylum) for each species used to calculate taxonomic diversity (Δ) and taxonomic distinctness (Δ*).
(XLSX)
