# Supplementary figures and images for: The Impact of Global Warming and Anoxia on Marine Benthic Community Dynamics: an Example from the Toarcian (Early Jurassic)
Source: PLoS One. 2013 Feb 14;8(2):e56255. doi: 10.1371/journal.pone.0056255 (PMC3572952; doi:10.1371/journal.pone.0056255)

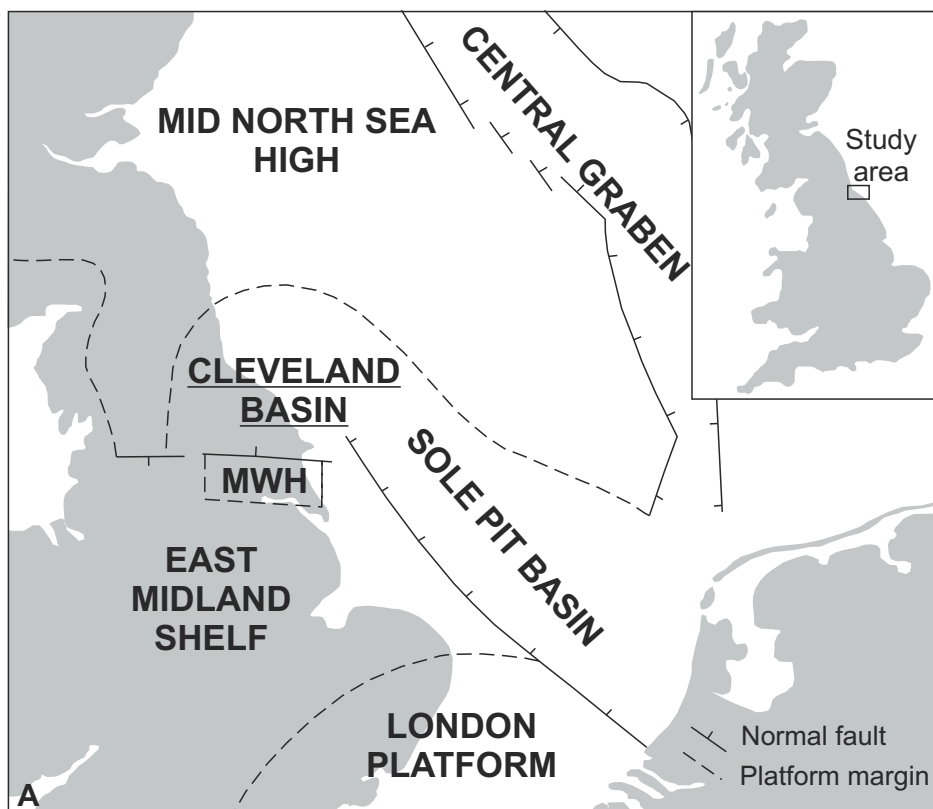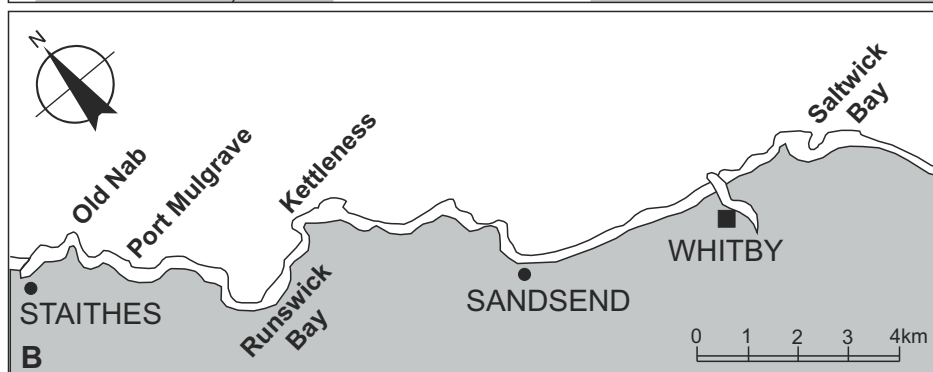

Supplement: Figure S1 — Location map of the study area. (A) Schematic structural map of the Cleveland Basin, modified from [50]. MWH: Market Weighton High. (B) Location of the studied sections on the North Yorkshire coast. (PDF) [file pone.0056255.s001.pdf]

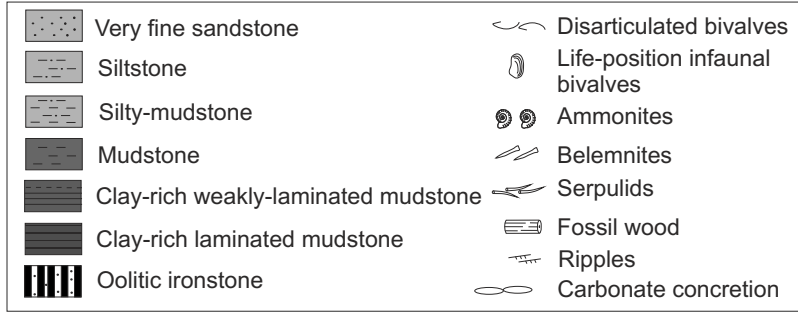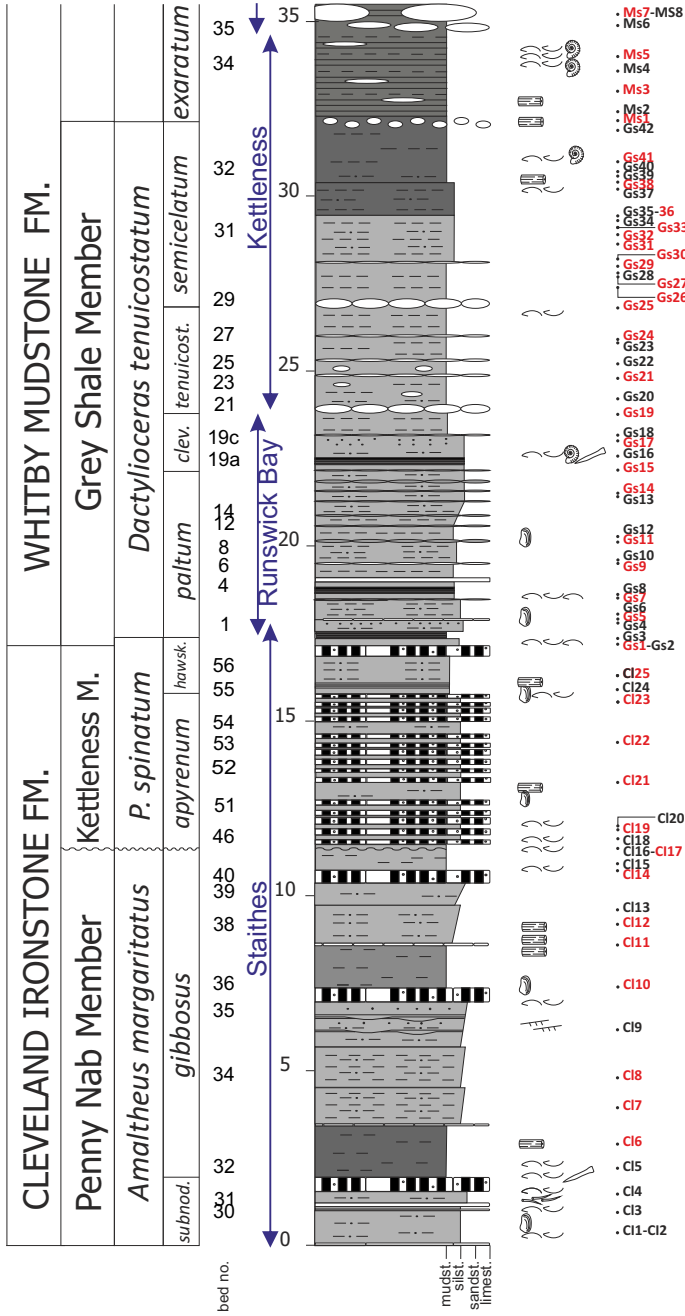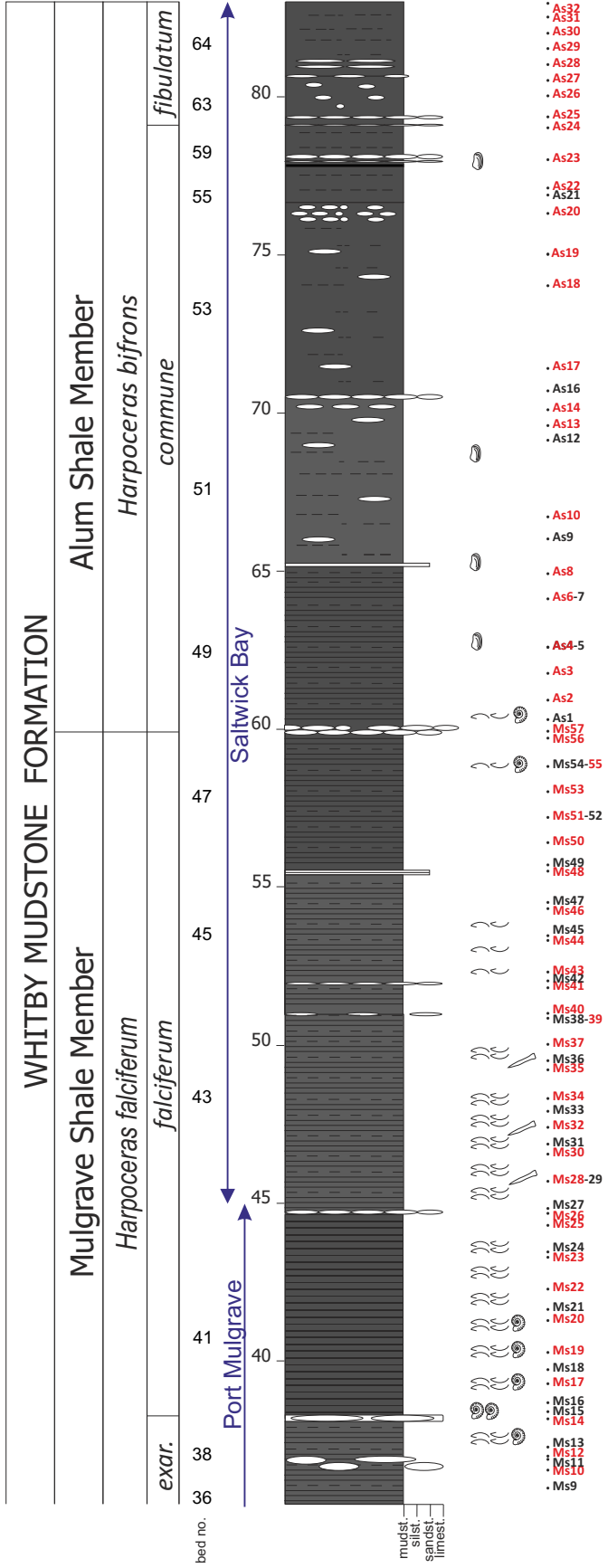

Supplement: Figure S2 — Detailed stratigraphic section. Stratigraphic log of the studied composite section with details of ammonite zones and subzones, lithology, bed numbers (following [55], [59], [62]), location of the main shell beds and of the collected samples and sampled localities (in blue). (PDF) [file pone.0056255.s002.pdf]

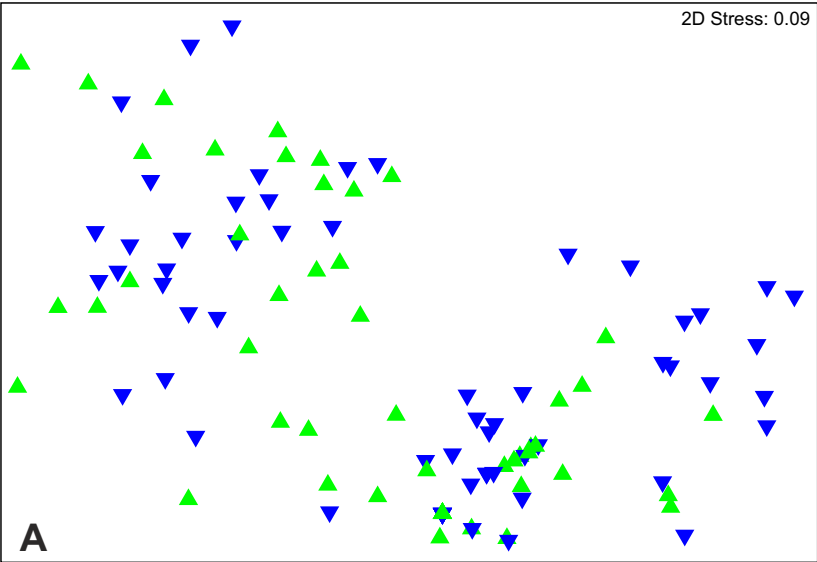

*Sampling methods*  
*nMDS Axes 1-2*

- ▲ SD-RJT-MEC
- ▼ CTSL

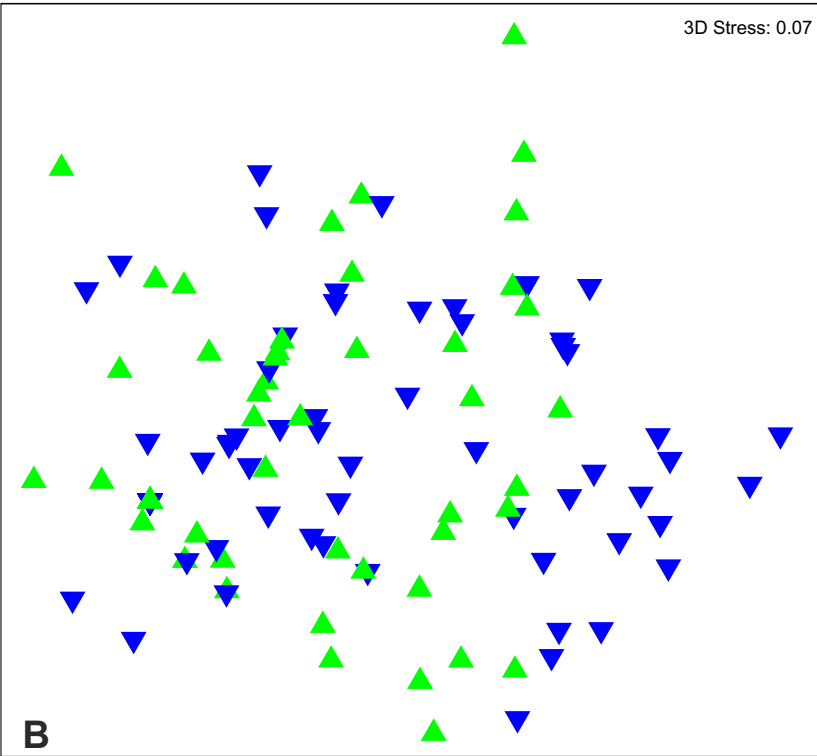

*Sampling methods*  
*nMDS Axes 2-3*

- ▲ SD-RJT-MEC
- ▼ CTSL

Supplement: Figure S4 — nMDS plots showing the distribution of samples collected with two different sampling methods. (A) nMDS ordination of axis 1 versus axis 2. (B) nMDS ordination of axis 2 versus axis 3. In green samples collected by SD, RJT and MEC; in blue samples collected by CTSL. Note, even though collected with two different sampling methods, the samples show a very high degree of overlap. This, together with the results of the ANOSIM test (R = 0.003, p = 0.324) discussed in the text, indicates that the dataset is homogenous. (PDF) [file pone.0056255.s004.pdf]
