# Supplementary material for: The Impact of Global Warming and Anoxia on Marine Benthic Community Dynamics: an Example from the Toarcian (Early Jurassic)
Source: PLoS One. 2013 Feb 14;8(2):e56255. doi: 10.1371/journal.pone.0056255 (PMC3572952; doi:10.1371/journal.pone.0056255)
Supplement: Figure S3 — Comparison of nMDS and DCA ordination of samples. (A) nMDS ordination with labels as samples numbers. (B) nMDS ordination with labels as bed numbers. (C) DCA ordination with labels as sample numbers. Note that nMDS and DCA produce similar clusters of samples, however the DCA ordination has a triangular shape derived by the compression of values on axis 2. (PDF) [file pone.0056255.s003.pdf]

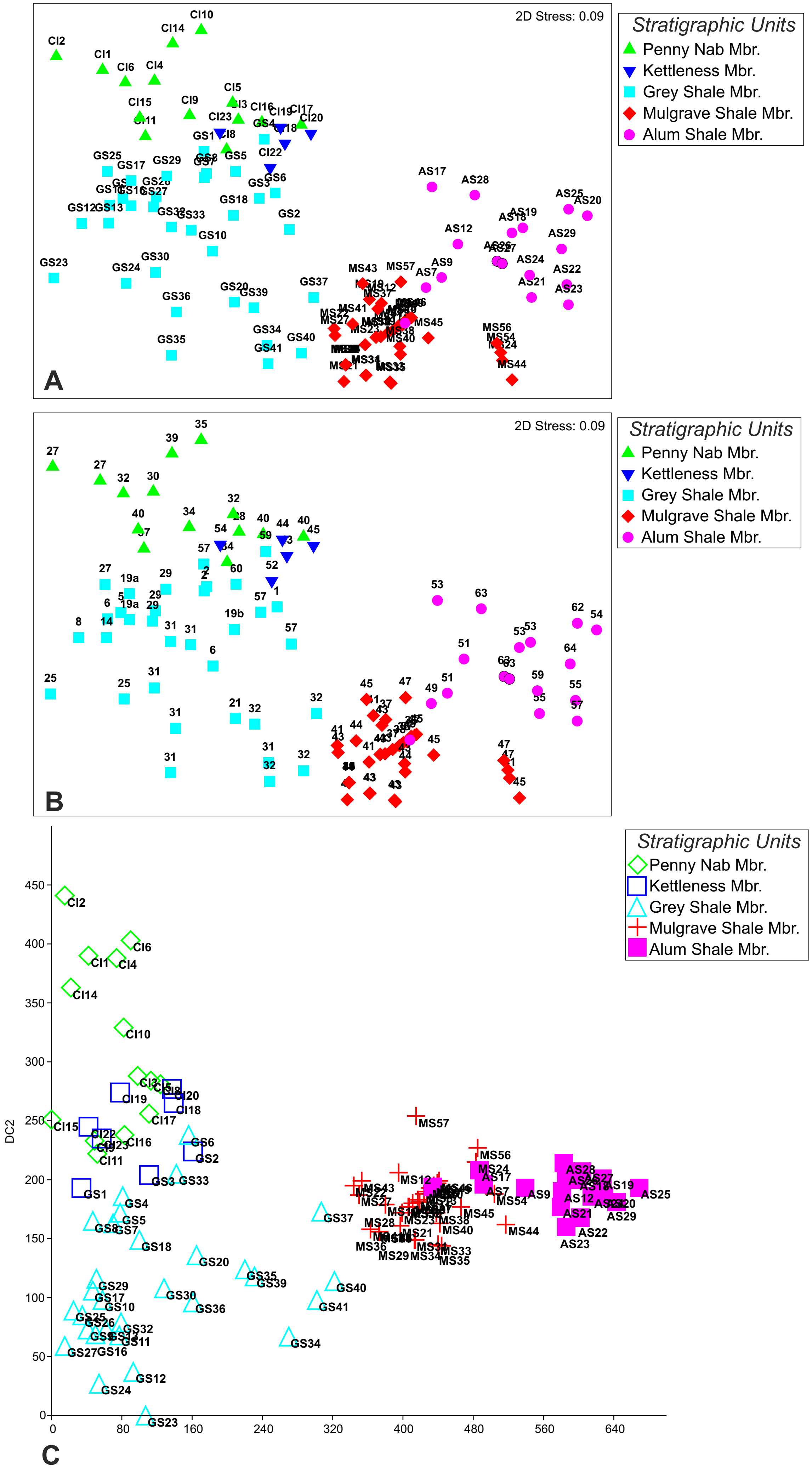
2D Stress: 0.09
Stratigraphic Units
Penny Nab Mbr.
Kettleness Mbr.
Grey Shale Mbr.
Mulgrave Shale Mbr.
Alum Shale Mbr.
A
2D Stress: 0.09
Stratigraphic Units
Penny Nab Mbr.
Kettleness Mbr.
Grey Shale Mbr.
Mulgrave Shale Mbr.
Alum Shale Mbr.
B
Stratigraphic Units
Penny Nab Mbr.
Kettleness Mbr.
Grey Shale Mbr.
Mulgrave Shale Mbr.
Alum Shale Mbr.
DC2
0
50
100
150
200
250
300
350
400
450
0
80
160
240
320
400
480
560
640
C
